# Risk Assessment for the Use of Drones in Warehouse Operations in the First Phase of Introducing the Service to the Market

**DOI:** 10.3390/s21206713

**Published:** 2021-10-09

**Authors:** Agnieszka A. Tubis, Jacek Ryczyński, Arkadiusz Żurek

**Affiliations:** 1 Department of Technical Systems Operation and Maintenance, Faculty of Mechanical Engineering, Wroclaw University of Science and Technology, Wyspianskiego Street 27, 50-370 Wroclaw, Poland; 2 Faculty of Security Sciences, Institute of Safety Engineering, Tadeusz Kosciuszko Military University of Land Forces (MULF), Czajkowskiego Street 109, 51-147 Wroclaw, Poland; jacek.ryczynski@awl.edu.pl; 3 Faculty of Technical and Economic Science, Witelon State University of Applied Sciences in Legnica, Sejmowa Street 5A, 59-220 Legnica, Poland; arkadiusz.zurek@gsuite.pwsz.legnica.edu.pl

**Keywords:** New Service Development, unmanned aerial system, risk assessment, warehouse operations, Logistics 4.0

## Abstract

Services, unlike products, are intangible, and their production and consumption take place simultaneously. The latter feature plays a crucial role in mitigating the identified risk. This article presents the new approach to risk assessment, which considers the first phase of introducing the service to the market and the specificity of UAV systems in warehouse operations. The fuzzy logic concept was used in the risk analysis model. The described risk assessment method was developed based on a literature review, historical data of a service company, observations of development team members, and the knowledge and experience of experts’ teams. Thanks to this, the proposed approach considers the current knowledge in studies and practical experiences related to the implementation of drones in warehouse operations. The proposed methodology was verified on the example of the selected service for drones in the magazine inventory. The conducted risk analysis allowed us to identify ten scenarios of adverse events registered in the drone service in warehouse operations. Thanks to the proposed classification of events, priorities were assigned to activities requiring risk mitigation. The proposed method is universal. It can be implemented to analyze logistics services and support the decision-making process in the first service life phase.

## 1. Introduction

Innovative technologies improve the quality of people’s lives by facilitating everyday activities through the careful design of products, services, and processes [1]. For this reason, the focus of the design process is the customer/user, their needs, and expectations. A typical user-centered design process has four significant steps [2]: (1) planning; (2) analysis; (3) creation; (4) verification. However, when analyzing the articles on the development of new products, it can be noticed that some authors describe the formal process of designing and introducing products to the market in 13 steps [3]. However, no matter how many steps the new product development procedure takes, its final stage is launching it on the market. When a product is introduced to the market, the hard work of the delivery team essentially comes to an end. However, in the development and introduction to the market of new services, the situation is precisely the opposite [4]. This is due to the different nature of the service and its strong dependence on the current conditions of performance.

The research proves that the service’s design, implementation, and development are different from the product’s case [5,6]. Among the four main features that distinguish a service from a product [4]:

1. Individualized experiences—each client receives the effects of the service individually. It means that the same service can bring different results in the assessment of individual clients.
2. Intangible asset—the fundamental aspect of the service is not a physical good (product). The customer receives real value and the emotions that accompany him when using the service.
3. Immediate assessment—customers’ reaction to the results obtained and the evaluation of the quality of the service provided is performed immediately (quick response time).
4. Inseparable elements—the service should be treated as a whole, being the sum of its parts.

Therefore, service development has its own set of attributes, challenges, and processes. Most of all, services are primarily immaterial, heterogeneous, and at the same time produced and consumed [7,8]. The last feature plays a crucial role in the activities undertaken concerning the mitigation of the occurring risk. The simultaneous production and consumption significantly impact the available risk management tools and often limit taking preventive actions related to selected adverse events [9]. At the same time, its non-material nature means that the customer experience is what is processed and re-evaluated by the customer after the service is completed.

For this reason, according to [4], the critical customer experience is the essence of the successful development of new services. For this reason, services must make a lasting, positive impression, meet customer expectations, and respond to their feedback to ensure implementation success. Activities aimed at mitigating adverse events that may occur during services are of fundamental importance.

The area of research is the use of an unmanned aerial vehicle system (UAV) in selected warehouse operations. According to [10], the UAV implementation aims to reduce operation times and eliminate human labor in repetitive, simple functions. One such area is the inventory process, which requires scanning numerous information carriers (barcodes or RFID tags) on goods located in the warehouse. This process is particularly labor-intensive in large high-bay warehouses, where tens of thousands of assortment items are stored. 

Not all enterprises require a daily inventory requirement in their activities. Most warehouses carry out this process periodically (monthly, quarterly, etc.). However, carrying out such an inventory is a severe challenge each time due to the need to suspend the operation of the warehouse and involve the entire warehouse staff in the inventory registration process. For this reason, the need to automate this process is increasing to shorten the time of its implementation and reduce human error. However, not all companies want to invest in their UAV system. This is primarily the lack of demand for a high frequency of such a measurement and the absence of a team of experts who adequately maintain and care for the development of such a system.

For this reason, there was a demand on the market for a service related to the use of drones in selected warehouse operations. However, the introduction of such a service provided in the customer’s work environment causes new undesirable events to occur in warehouse operations, especially in the first phase of its life cycle. This requires special preparation of the service team and limiting the potential risk if the company wants to succeed in the market. Determining the risk level will also lower the possible losses resulting from disruptions occurring during the service performance. As a result, the efficiency of the development process will increase, which, according to the research of Storey et al. [5], is one of the ten success factors of New Service Development (NSD).

According to many authors, risk assessment and mitigation is perhaps the most critical activity in any NSD project [11,12,13]. At the same time, however, Hsieh et al. [14] prove that the assessment for these projects is often not adequately carried out. This limits the success of NSD due to the lack of a sufficiently quick response to adverse events, especially in the early stages of the service life cycle. For this reason, the article aims to propose a new approach to risk assessment, which considers the first phase of introducing the service to the market and the specificity of the use of UAV systems in warehouse operations. The main contributions of this study are:

- Development of a risk assessment method for NSD, taking into account the use of the UAV system in logistics operations and the early stage of introducing the service to the market;
- Identification of possible disruptions occurring during the use of drones in the warehouse inventory process based on the research of the actual system at the early stage of introducing the service to the market;
- Implementation of the proposed approach to risk assessment and verification of the results obtained in natural conditions;
- Identification of the existing limitations of the proposed approach and barriers to its implementation.

The remainder of this article is structured as follows. Section 2 presents a literature review on the most critical issues related to the submitted research results. Then, Section 3 offers the proposed approach to risk analysis for using the UAV system in logistics operations. The proposed method has been implemented for the selected service. The assumptions made for this implementation are presented in Section 4 and the results obtained in Section 5. Section 6 presents the results of the discussions and indicates the areas for possible development of the proposed approach. At the end of the article, Section 7 presents the conclusions of the conducted research. 

## 2. Literature Review

The presented research method was developed based on industrial research and a literature review in four primary areas. The results of the literature studies made it possible to define the most critical guidelines for the developed methodology and to define the current directions of analogous research conducted by authors around the world. The essential characteristics of the research concepts taken into account in the developed approach are presented below.

### 2.1. New Service Development—NSD

Services play an essential role in the economy of developing countries. The continuous increase in the importance of the service sector and the increasing expenditure on research into innovations in this area resulted in the intensive development of new services over the last decade [6]. Effective development of new services is vitally important, primarily because it influences the success of the resulting service product and the company’s competitive position [15,16,17,18]. Storey et al., in their research, indicate the top ten success factors for NSD, which include [5]: (1) Launch proficiency; (2) Absorptive capacity; (3) Organizational design; (4) Innovation strategy; (5) Efficiency of the development process; (6) Service innovativeness; (7) Front-line staff involvement; (8) External relations; (9) Internal communication; (10) Formal/structured development. However, many authors research the identification of critical success factors. Many of them indicate in their results that the service must be helpful, accessible, and demandable by customers, as well as an intention to be effective, efficient, and different from the competitors [19,20,21]. Noteworthy is the classification of critical success factors in NSD, proposed by Kitsios and Kamariotou [22], based on the analysis of 144 articles in this area.

Griffin [23] notes in his research that only 58% of new services are successful. In his results, Alam [24] indicates the lack of a properly prepared strategy as the failure to introduce new services to the market. In his opinion, they develop unsuccessful service companies because they do not understand customers’ needs and do not listen to their insights. For this reason, they are unable to create their new services strategically. Customers play a direct role in providing services, so their involvement in the development process is more important than developing new products [4]. This is also confirmed by the research of Lievens and Moenaert [25]. They indicate that due to the non-material nature of the service, their proper development requires intensive information exchanges between service employees and customers.

For the abovementioned reason, the team providing the service plays an important role. These employees have direct contact with customers and receive feedback from them about the service. This provides them with an excellent reference point for identifying unmet needs and frustrations. As noted by Kahn et al. [4], the involvement of this staff is necessary, especially in the early stage of identification of problems and threats. Employees of the service delivery team can be an excellent source of adverse event hypotheses and identifying areas of frequent or intense customer dissatisfaction with the service provided.

As indicated in the Introduction, the design, implementation, and development of the service are different from the same processes carried out in the case of the product [5,6]. While the overall New Service Development strategy should reflect the method used to develop new products, service development requires a unique, hands-on approach due to service delivery’s complex, diverse nature [4]. Some authors (e.g., [5]) even indicate that innovative product development may be inappropriate for services. For this reason, many researchers focus their activities on developing the structure for many activities and concepts related to the NSD process [26,27]. In this case, the model developed by Johnson et al. [28] synthesized past service development research and created a general four-stage NSD process model involving the phase of design, analysis, development, and full launch.

In the model we are describing, the area of interest is primarily this last stage of full launch. However, information on the earlier stages of service development are not without significance. Their course and the results may affect the risks in the first phase of introducing the service to the market. It should be remembered that while introducing the product to the market, the hard effort of the development team ends; in the case of services, it is precisely the opposite [4]. The service requires constant monitoring and evaluation to respond to customer needs most efficiently and effectively throughout its life cycle. There is always space for improvement in the implementation of the service, which is why constant feedback from customers is essential. Kahn et al. [4] also emphasize that it is more difficult to predict the receipt of a new service and the course of its implementation compared to the delivery of a new product. While it is possible to test a service and a product, external conditions often significantly influence a customer’s assessment of the value of a service. It should also be emphasized that the simultaneous production and consumption of the service may cause heterogeneity in its implementation. This is mainly due to the inconsistency in human performance [29] and relies on the people’s tacit knowledge [5]. Many of these factors may be unpredictable before the service is introduced to the market, so much importance should be attached to improving the service in the first period of its delivery to customers. Identifying the key variables that affect the quality of service will allow better identification of adverse events which should be subject to risk assessment in the first phase of introducing the service to the market.

### 2.2. Unmanned Aerial Vehicle in Logistics 4.0

The concept of Logistics 4.0 is an integral part of the Fourth Industrial Revolution known as Industry 4.0. Wang [30] defines Logistics 4.0 as a collective term for technologies and concepts of value chain organization. Cyber-physical systems monitor the processes of physical material flow, create a virtual copy of the physical world, and make decentralized decisions. Szymańska et al. [31] note that when talking about Logistics 4.0, it should be taken into account that this concept combines two aspects: processual (supply chain processes are a subject of the Logistics 4.0 actions) and technical (tools and technologies that support internal processes in the supply chains. Wawrla et al. [10] emphasize that the Fourth Industrial Revolution in the area of Logistics 4.0 applies, in particular, to warehouses. This situation results from the fact that in warehouse processes, new technologies related to automatic data identification (bar codes, QR codes, radio frequency identification (RFID)) and autonomous vehicles (autonomous mobile robots and unmanned aerial vehicles) appear in the first place. The use of autonomous vehicles in this area results primarily from the desired increase in reliability for the operations performed, and as noted by Bechtsis and Tsolakis [32], from the expected economic benefits associated with:

- the capability to function on a 24/7 basis;
- the minimization of labor cost;
- the low maintenance cost;
- the enhanced accuracy in daily activities;
- the improved safety at industrial facilities.

The research presented in the article focuses on the use of drones in the Logistics 4.0 concept. Currently, many authors (e.g., [33]) argue in their research that unmanned aerial vehicles are a key technology in smart factories. This situation results from the possibility of their use in repetitive and dangerous tasks in which human participants should be limited. This is also confirmed by the publications in which the wide use of drones is described in fields like remote sensing (e.g., mining), real-time monitoring, disaster management, border and crowd surveillance, military applications, delivery of goods, precision agriculture, infrastructure inspection or media and entertainment, among others [34,35]. As evidenced by the research presented in [36], the use of unmanned aerial vehicles in Logistics 4.0 will represent future trends in the next five years. Additionally, Wawrla et al. [10] emphasize in their research that the growing popularity of drones is due to their ability to fly and hover autonomously, avoid obstacles in different warehouse layouts, navigate indoors, and land precisely. This is also because, unlike any ground machine, the drone’s movement is not limited to the ground but extends to all three dimensions [37]. 

Wawrla et al. [10] distinguished three warehouse processes with the highest level of drone use. These processes are presented in Table 1.

Examples of new initiatives and projects related to drones in warehouse processes can be found primarily in industry publications and research reports. The research presented by Deepak [37] should be highlighted here. The goal was to develop an automated system to perform routine warehouse inventory processes without any human interference. An interesting initiative is a cooperation between Geodis and Delta Drone [38], which developed a fully automatic drone warehouse inventory solution. The main benefits of implementing this solution are indicated by [38]: the productivity gains generated by the realization of the inventories outside the hours of activity of the warehouse, the reinforcement of the safety at work for the collaborators of the site which no longer have to perform this tedious and sometimes risky task, and reliable inventory management. In the project presented by start-up Ware [39], the authors proved the economic benefits of using drones based on selected key metrics: accuracy, number of total inventory counts, lost inventory, and headcount (the number of your needs on your inventory team). The obtained results proved that a group of humans, on average, covers 13 bins per hour, and each Ware drone can cover 75 bins per hour [39].

The use of drones in warehouse operations also generates many challenges. Maghazei and Netland [40] distinguish five generic categories of challenges and drawbacks related to the use of drones: (1) technological challenges; (2) operational challenges; (3) organizational challenges; (4) legislative challenges; (5) societal and mental challenges. However, it should be emphasized that even though the significant challenges to the industrial application of drones are related to technological limitations, the organizational challenges associated with this solution are no less important. The authors of many publications (among others [41]) prove that workers’ knowledge and technical experience, training, and involvement in planning are critical determinants for the success of technology adoption. Therefore, both of these categories should be the subject of specific research in the risk assessment process.

### 2.3. Risk Assessment in Logistic Services

In recent years, we have observed a growing interest in the concept of risk management and its use in the area of logistics services and supply chains. The idea of risk presented in the literature takes into account two dominant approaches [9]:

- approach 1—identifying the risk through the prism of the occurrence of adverse events;
- approach 2—interpreting risk as events affecting negative (disruptions) and positive (opportunities).

Concerning anthropotechnical systems, approach 1 is primarily taken. This is confirmed, among others, by the extensive literature review presented by Aven [42]. Additionally, approach 1 dominates in research on risk in supply chains and logistics systems. For this reason, in the proposed methodology for risk assessment for NSD, which concerns the use of UAV systems in logistics operations, this approach to risk has also been adopted as obligatory. For this reason, the entire proposed procedure is focused on the risk of adverse events occurring during the performance of the service.

Logistical risks have been considered as an essential category of risks faced by firms [43]. Many authors consider logistics risk management as a subsystem in the supply chain risk management system. For this reason, many publications distinguish between two sources of risk—internal and external (see Table 2). Internal risk is usually related to the company’s internal logistic processes. In contrast, the external risk is related to the company’s functioning in a specific business environment created within a given supply chain and environment.

When analyzing the risk associated with logistics processes, the authors focus primarily on the processes related to transportation, storage, and inventory [48]. For this reason, the most common scenarios for risk assessment in logistics processes include [49,50]: warehousing and production interruption; lack of information transparency; damages in transport; unplanned machine stoppages; serious forecasting errors, and poor quality of raw material.

Many organizations outsource entire or some parts of the logistics activities, which leads to the emergence of external logistics service providers who participate in internal processes of the enterprise [51]. Some authors emphasize that including an external service provider in the logistics processes may generate additional risks in these systems [52]. Govindan et al. [43] indicate some risks associated with using services provided by 3PL:

- Disruption to inbound flow;
- Inadequate provider expertise;
- Inadequate employee quality;
- The inability of 3PL providers to deal with special product needs and emergency circumstances;
- Incompatibility of information systems between shipper and 3PL;
- The failure of 3PL to meet a shipper’s future growth needs;
- Lack of security.

The inclusion of external service providers in internal logistic processes causes a change in the characteristics of the occurring adverse events. The existing internal risks, which are under the control of the enterprise, become an external risk (generated by an external organization), over which the level of control is limited [9].

In the literature, various risk assessment approaches and methods are dedicated to logistics processes [53]. They can be used in subsequent stages of the analysis, and their selection usually depends on the type of data at the disposal of the researchers. The analysis methods described in the publications can be classified into one of three groups:

- Qualitative techniques [54];
- Quantitative techniques [55,56];
- Hybrid modeling combines quantitative and qualitative techniques [57,58,59,60,61].

One popular hybrid method used in various research and decision support models is fuzzy logic set theory [54,59,60,62,63,64]. In our proposed approach, we also decided to use this concept when estimating risk parameters. We used a fuzzy inference system, in which fuzzy rules built on expert knowledge and fuzzy logic support the natural language modeling process. Additionally, fuzzy expert systems provide an easy way to deal with situations involving fuzzy sets, both linear and uncertain properties [65]. The results are based on quality assessment, causal relationships, and impact analysis in these proceedings [66]. 

## 3. Methodology

Risk analysis in the early phase of introducing a service to the market plays a vital issue. It allows the company to identify the sources of existing threats to the effective and efficient service provision and enables its performance to be improved and its competitiveness to increase. The results of the obtained risk assessment should let the service provider take preventive actions that will reduce the likelihood of a given undesirable event and develop emergency scenarios allowing for a quick response and limiting the consequences in the event of a given disruption. For this reason, a significant role is played by the recording of any disruptions and the accompanying conditions occurring during the service implementation. The most critical data that should be included in the report after the service has been performed should include:

- Description of the primary conditions of the UAV system operating environment during the service provided to the customer (including the type of warehouse, area, number of racks, rack height, type of information carrier, type of lighting, humidity, and ambient temperature);
- Identification of the disturbance/undesirable event that occurred during the drone mission;
- The frequency of the disturbance during the implementation of the entire service (as part of one service, the drone usually performs from several to a dozen or so missions);
- The causes of the disturbance (if it is impossible to identify the causes—description of the conditions for the implementation of the mission during the disturbance occurrence);
- The consequence of the disturbance.

The collected data from individual drone missions should feed into the knowledge base created by the service provider. This database is built to improve the offered service and prepare a risk assessment. The team leader or the person responsible for recording this data should regularly update this database, preferably each time the service is performed. Thanks to this procedure, it is possible to periodically update the conducted risk analysis and its monitoring following the guidelines of ISO 31000: 2018. On this basis, it will be possible to introduce a risk management strategy to improve the service continuously.

**Figure 1:**
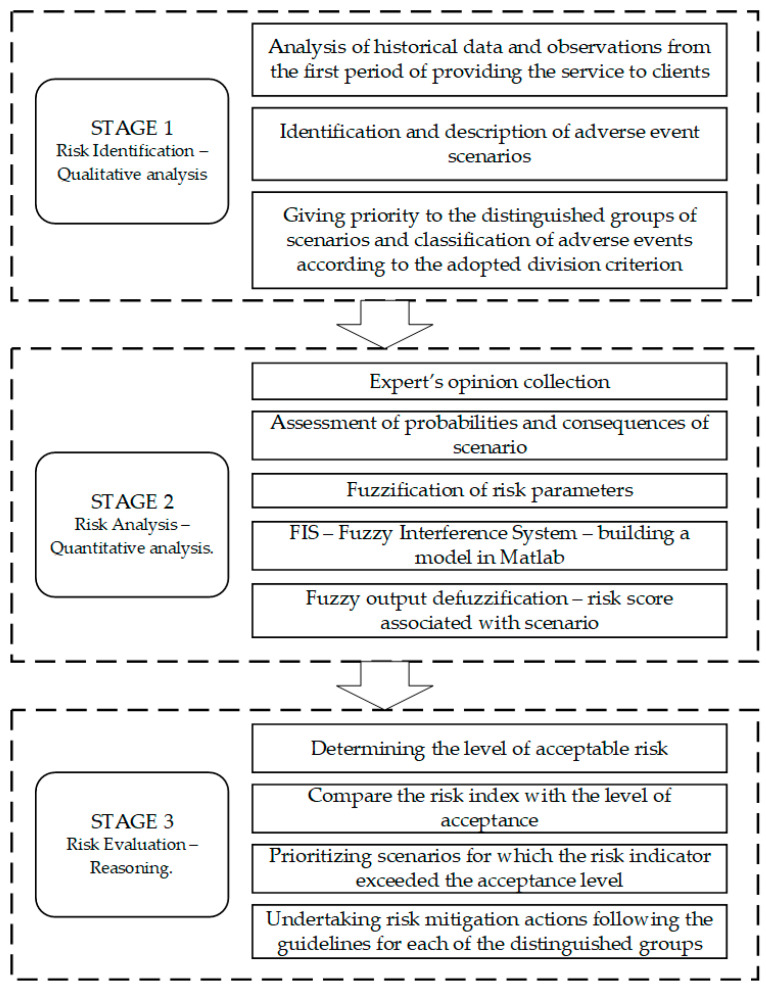
Procedure stages in risk assessment.

According to ISO 31000: 2018 [67], a risk assessment should include three stages of the procedure: (1) risk identification, (2) risk analysis, (3) risk evaluation. We have also adopted such a structure in the approach we propose, presented in Figure 1. 

### 3.1. Risk Identification—Qualitative Analysis

The risk assessment concerns scenarios of adverse events, the occurrence of which should be eliminated or limited during the provision of the service at the client’s premises. The basis for their identification is information on disturbances occurring in the initial period of introducing services to the market, which have been registered in the knowledge base. Each scenario should be described taking into account the causes of the undesirable event and its consequences. The 5 × WHY or SWIFT method and the traditional Brainstorm method can analyze the causes of the risk. However, to study the effects of the disturbances, it is recommended to use the What-If method. The conducted cause-and-effect analysis should enable the process of classification of scenarios according to the adopted division groups. 

In the first phase of introducing a service to the market, it is necessary to group the assessed scenarios properly at the risk identification stage. The purpose of the classifications is to react faster to emerging threats and better adapt risk management tools to the current needs. Giving a rank/priority to selected groups of scenarios will allow considering their importance in improving the service, increasing its market competitiveness, or limiting significant losses. At the early stage of introducing the service of using drones in warehouse operations to the market, it is proposed to distinguish two groups of scenarios presented in Table 3.

Concerning the analyzed service, identifying disruptions caused by incorrect operation of the UAV system is of particular importance. Despite testing the devices used, in the first stage of introducing the service to the market, certain disturbances related to the operation of the technical elements of the system are allowed. Of course, it is crucial to quickly identify and eliminate them as part of improving the entire measurement system. For this reason, such a key role is played by a properly conducted risk assessment already at the initial stage of introducing the service to the market. It should be remembered that the lack of corrective actions on the part of the service provider will translate into the repetition of the undesirable scenario, low quality of service assessment by subsequent customers, and even increasing losses related to the unsatisfactory level of the service provided. Therefore, these scenarios should be assigned a higher rank in the risk management process. 

The drone application service is carried out in the work environment created by the customer in his warehouse facility. Therefore, the working conditions of the drone are variable and depend on the conditions in the warehouse and on the customer’s preparation. Thus, in the process of providing a service, not all factors influencing its performance remain under the control of the service team. Disruptions resulting from this group of scenarios are of less importance (lower priority). They remain beyond the company’s control and therefore have a minor impact on the customer’s service assessment. Of course, disruptions caused by factors beyond the control of the service provider should also be mitigated. They can be limited by introducing appropriate procedures for preparing the working environment by the client and contingency scenarios in the event of a given scenario. Therefore, they must also be subject to a risk assessment and monitoring process.

As indicated in [4], in identifying problems early in the life of a service, it is necessary to involve the supplying staff. They participate in providing the service and have direct contact with the client, thanks to which they know his opinions, complaints, and needs. Additionally, in the proposed approach, it is recommended to include members of the teams providing the service at the client’s site in the risk identification process. Their knowledge and experience are precious in investigating the causes of identified adverse events and assessing the consequences of their occurrence. For this reason, members of service teams should actively participate in determining the grounds of the occurring scenarios because their point of view is highly analytical. Thus, each of them contributes their comments to the various stages of the service implementation. In contrast, team leaders should be part of a team of experts assessing the impact of these events. Leaders have a broader view of the entire service process and can evaluate the occurring phenomena synthetically.

### 3.2. Risk Analysis—Quantitative Analysis

The risk in the analyzed scenarios has been described by the Kaplan and Garrick approach, which was defined as follows [68]:

(1) $$R=\left\{S_{i},\;P_{i},\;C_{i}\right\},\;i=1,\;2,\;\ldots ,\;N$$

where:

*R—*risk;

{}*—*must be interpreted as a “set of”;

*S—*a scenario (undesirable event) description;

*P—*likelihood (probability) of a scenario, expressed by the frequency of its occurrence;

*C—*the measure of consequences or damage caused by a scenario;

*N—*the number of possible scenarios.

There is one detail in Equation (1) to be explained. “Likelihood” (*P* in the equation) does not mean the classical mathematical probability, defined in the range from 0 to 1, but the probability of a scenario, expressed by the frequency of its occurrence. The term “likelihood” means a linguistic probability ranging from 1 to 10. Only adopting such a range enables the correct analysis of the problem because the identical range was adopted for consequences of scenario occurrence.

In the initial phase of constructing the model, the authors chose triangles as the leading shape of the membership function (MF) at the input. However, such a choice resulted in generating “inactive” areas on the surface of risk scores, which was the result of the model’s operation (horizontal fields with a constant value of the Risk Level index). Additionally, there was an abrupt change in the Risk Level index when moving between successive levels. The analysis of the historical research results during services performed at the clients’ premises and the obtained results of the model’s operation in MATLAB (version R2021a) determined that Gaussian curves would represent input membership functions. The obtained results confirmed the correctness of this approach. It is essential to mention how the Fuzzy Logic Designer (FLD) module works in MATLAB. This module does not allow users to interfere with the calculation algorithm.

The operator’s action is limited to selecting a computing system (SUGENO or MANDANI), defining a membership function and defining rules. This has a noticeable impact on the obtained test results. In addition, we should pay attention to one more feature of the FLD module itself. In SUGENO systems, the output range is uniquely defined by the range and type of input membership functions. Therefore, the choice of the starting range does not affect the course of the surface. This approach should be considered a limitation for SUGENO systems as the output data are not fuzzy sets. For systems using the MAMDANI algorithm, the output depends on both the input and output MF because both inputs and outputs are fuzzy sets. The output signal range should typically be selected to contain the cumulative values of the minimum and maximum values of all output MF. A comparative analysis of the research results obtained using two different computational systems will also be the subject of future research by the authors. To confirm the correctness of the MF input choices, the Results section, apart from the control surface plot, also presents the dependence of individual model input signals on the obtained output Risk Level index results.

To correctly determine the probability and impact of each scenario, direct interviews were conducted with a group of experts who were, directly and indirectly, involved in the planning, organization, implementation, and supervision of the proper functioning of the customer’s inventory process. Linguistic variables were used to gather expert opinions on the parameters assessed. Then, these variables were modeled using the fuzzy set theory. According to this theory, the risk parameters of each scenario (in the case of the problem being solved: consequences and the level of risk) are treated as a fuzzy number (FN) and the membership function (MF) is related directly to it. Membership functions can take many shapes. The choice of the body for a specific fuzzy set (language value/attribute) is subjective and depends on the problem being solved. The presented risk assessment method proposes the use of MF Gauss (probability of the scenario and consequences of the scenario) and the trapezoidal MF (level of risk).

A Gaussian FN is presented by a doublet *A_z_* = (*a*, *b*) and its MF is given by (2):

(2) $$\mu_{z}\left(x\right)=e^{-{\left(\frac{x-a}{b}\right)}^{2}},\;\;\;x\epsilon R,\;\;b>0$$

**Figure 2:**
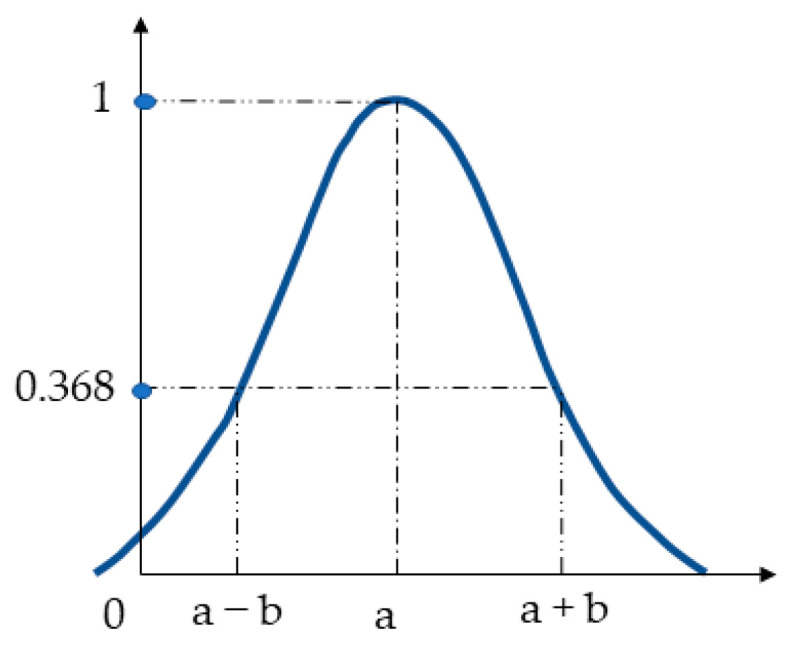
The shape of the membership function of a Gaussian fuzzy number.

Graphic interpretation of Gaussian fuzzy number and its MF is shown in Figure 2.

Trapezoidal FN, defined as *A_z_* = (*a*, *b*, *c*, *d*), has its MF given by (3):

(3) $$\mu_{z}\left(x\right)=\{\begin{array}{l} 0\;\;\;\;\;\;\;\;\;\;\;\;\;\;\;\;\mathrm{for}\;x<a\\ \frac{x-a}{b-a}\;\;\;\;\;\;\;\;for\;a\leq x\leq b\\ 1\;\;\;\;\;\;\;\;\;\;\;\;for\;b\leq x\;\leq c\\ \frac{d-x}{d-c}\;\;\;\;\;\;\;\;for\;c\leq x\leq d\\ 0\;\;\;\;\;\;\;\;\;\;\;\;\;\;\;\;\;for\;x>d \end{array}$$

The meaning of the MF parameters is straightforward—a and d are the lower and upper bounds of fuzzy number *A_z_*, respectively, and b and c are the modal value of fuzzy number *A_z_*. 

Expertise (expert opinion) is the primary tool for conducting aggregate operations by which the level of risk is obtained. One of the methods [69] is to use the arithmetic mean aggregation operator. Aggregation represents an operator defined in Gaussian FN (*a*_1_, *b*_1_), (*a*_2_, *b*_2_) … (*a_n_*, *b_n_*); *n* is a number of experts, delivers the result as (*x, y*) according to the Formulae (4):

(4) $$\{\begin{array}{c} x=\frac{1}{n}\sum_{k=0}^{n}a_{k}\\ y=\frac{1}{n}\sum_{k=0}^{n}b_{k} \end{array}$$

**Figure 3:**
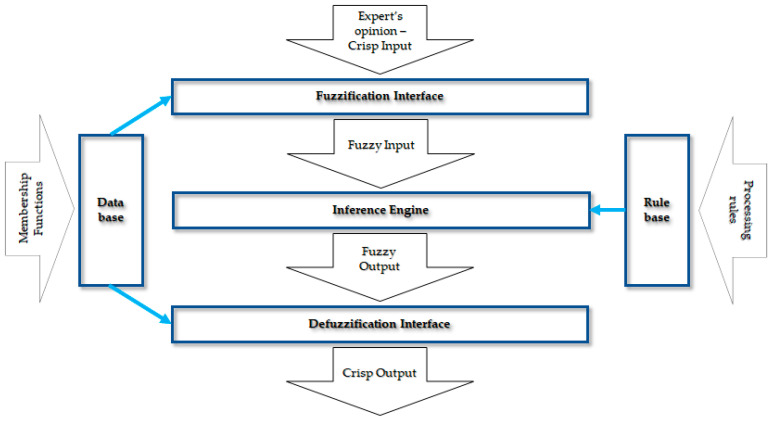
MAMDANI fuzzy model in the analyzed research problem.

The following steps in this phase are to quantify the risk. This process is based on the use of the MAMDANI fuzzy model [70]. The MAMDANI fuzzy interference mechanism is based on the method proposed by Zadeh [71]. According to this proposal, there are four main modules in Mamdani fuzzy model: Fuzzification, Knowledge base, Fuzzy Interference System, and Defuzzification [72]. Figure 3 presents a diagram of the MAMDANI model for the research problem to be solved.

As mentioned, fuzzification process is based on Gaussian FNs. The Gaussian FN transforms linguistic scales in the 0–1 range by using its MF.

**Figure 4:**
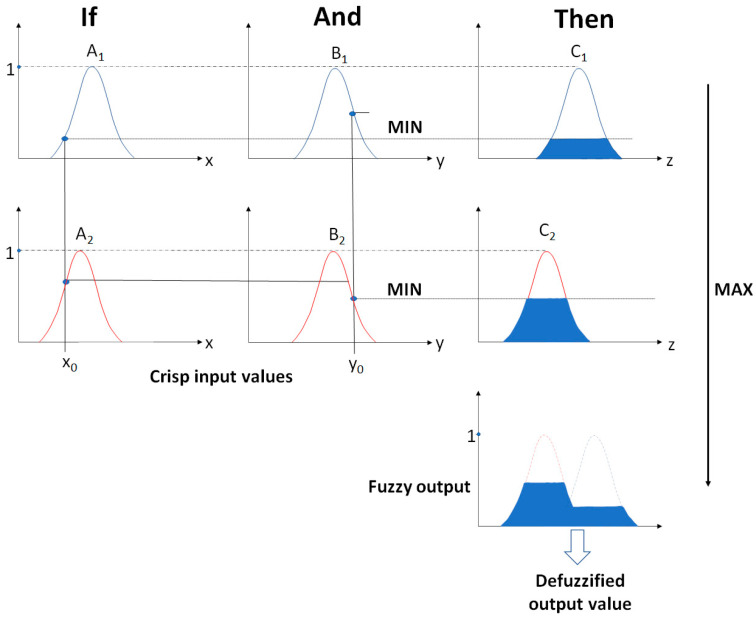
Functioning of FIS of MAMDANI model on the example of Gaussian curves using MIN and MAX operator.

The system is designed to map fuzzy inputs and control the values obtained on the outputs using the fuzzy set theory. The FIS of the MAMDANI fuzzy model uses the action of two types of operators: the so-called MIN and MAX operators. The MIN operator is used in combination and implication operations. In turn, MAX is used for fuzzy aggregation results. The functioning of the FIS with the use of the described operators for the research problem being solved is presented in Figure 4.

The last stage of the proposed model is the process of defuzzification. Its purpose is to convert the fuzzy output into a crisp output signal. Thus, the method of defuzzification of the centroid of area, is following the research results given, for example, in [73,74]. The output value is determined by Equation (5) [72]:

(5) $$\mathrm{Centroid}\;\mathrm{of}\;\mathrm{area},{\;z}^{*}=\frac{\int^{\;}\mu_{A}\left(z\right)\times \mathrm{zdz}}{\int^{\;}\mu_{A}\left(z\right)\mathrm{dz}}$$

where:

z*—defuzzified output;

$\mu_{A}\left(z\right)$—the aggregated output MF;

z—the universe of discourse.

The effect of defuzzification is a clear output value entering the output phase.

### 3.3. Risk Evaluation—Reasoning 

The last phase of the risk assessment process is its evaluation. At this stage, the risk acceptance level is determined, which is the basis for future risk management activities. The level of acceptance depends on the risk perception of decision-makers and is often subjective. For this reason, it should be developed by a team of experts who have diversified knowledge, experience, and other factors that may affect their risk perception. Thanks to a mixed team, a certain level of acceptance will result from a particular compromise and not take on extreme values.

In the proposed method, it is suggested to consider the early phase of the service life cycle and determine the acceptance level. This means that the team of experts accepts a lowered level of acceptance, which will allow for a faster response to any disruptions. Thanks to this, it will be possible to improve the process and the UAV system at an early stage of the assessed risk, which will reduce the potential effects of its occurrence. As described in Section 3.1, the priority given to a given group of scenarios is also considered when determining the acceptance level. This way, higher priority scenarios will have a lower acceptance rate. This will enable faster actions to be taken to improve the service and eliminate the sources of interference. On the other hand, scenarios with a lower priority will have a higher level of acceptance to take practical actions to improve the quality of the service provided.

The acceptable risk level is the basis for the scenario classification process regarding actions taken to reduce and monitor it in subsequent periods. For scenarios whose risk index exceeds the defined acceptance level, it is necessary to take risk-mitigating measures. The scope of these activities and the implementation priority assigned to them depend on the classification of the scenario into one of the groups distinguished in the first phase of the risk assessment. 

For scenarios whose estimated risk index is within the accepted level of acceptance, no mitigating actions are taken. However, it should be taken into account that the early phase of service life requires continuous monitoring of the service process and a quick response to emerging threats. For this reason, even for low-risk scenarios, it is necessary to update its current level, which will allow for an immediate reaction of decision-makers in the event of an observed increase in the value of one of the estimated risk parameters. 

## 4. Application of the Proposed Approach

The proposed approach was implemented in a selected company, which, in 2020, introduced the service of using drones to inventory on the market. Among the customers using the company’s services, there are, among others, logistics operators, producers, and local government organizations. The risk analysis was carried out based on data regarding disturbances registered for the services provided from 1 March 2020 to 28 February 2021. During this time, the service team carried out over 300 drone missions in high-bay warehouses (max. height 12 m) with various customers (various conditions of the system’s operating environment).

Based on the recorded disturbance data, ten adverse event scenarios were identified during the measurements, which were the subject of further analysis. The characteristics of these events and their causes are presented in Table 4.

**Figure 5:**
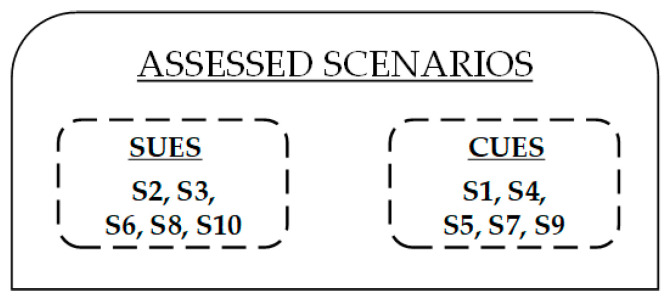
Classification of assessed scenarios.

Identification of the reasons for the occurrence of each scenario is the basis for its correct classification to the distinguished groups. For this reason, the identification process was carried out in collaboration with service team members who participated in the missions for which disruptions were recorded. The 5 × WHY and Brainstorm methods were used for the analysis, making it possible to quickly identify the causes of the disturbances and classify the scenario to the appropriate group: SUES or CUES. The results of the performed classification are shown in Figure 5. 

Information on the effects of each of the analyzed scenarios was obtained from the collected historical data. On their basis, it was possible to distinguish four primary groups of effects:

- Damage or crash to the drone;
- Unable to start/complete the mission by the drone;
- Lack of expected effects of the completed task;
- Delays in completing the assignment by the drone.

On the basis of the abovementioned and based on the results of the What-If analysis carried out by experts, the possible effects of adverse events have been expanded to five levels presented in Table 6.

A team of experts with knowledge and experience in UAV systems and their implementation in logistics systems was appointed to estimate the probability and effects for each scenario. To ensure the high quality of the analysis, the experts had different competencies, knowledge, and experience. The group consisted of representatives of:

- The scientific community (researchers from universities);
- Service team leaders (responsible for the performance of the service);
- Engineers (responsible for the operation and development of the UAV system used);
- The representative of the INTL Institute (a company specializing in improving logistics processes, including UAV systems).

Based on their opinions, linguistic descriptions were developed for both assessed parameters and the size of the estimated risk.

The assessed risk concerns the service in its early stage of introduction to the market. Therefore, the number of measurements feeding the knowledge base with disturbance data is limited. This fact forces a more restrictive approach to estimate the probability of occurrence of individual events. For this reason, when defining linguistic variables for the estimated probability of the occurrence of particular scenarios, the experts adopted a disproportionate range of the adopted degrees of the assessment scale used. The likelihood of a scenario expressed by the frequency of its occurrence is presented in Table 5.

**Figure 6:**
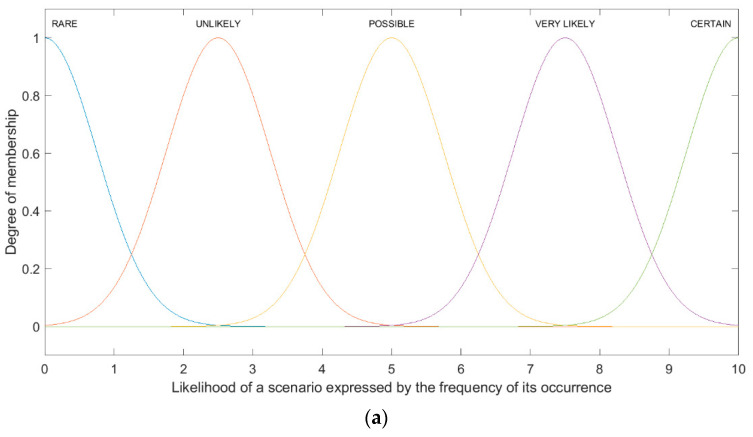

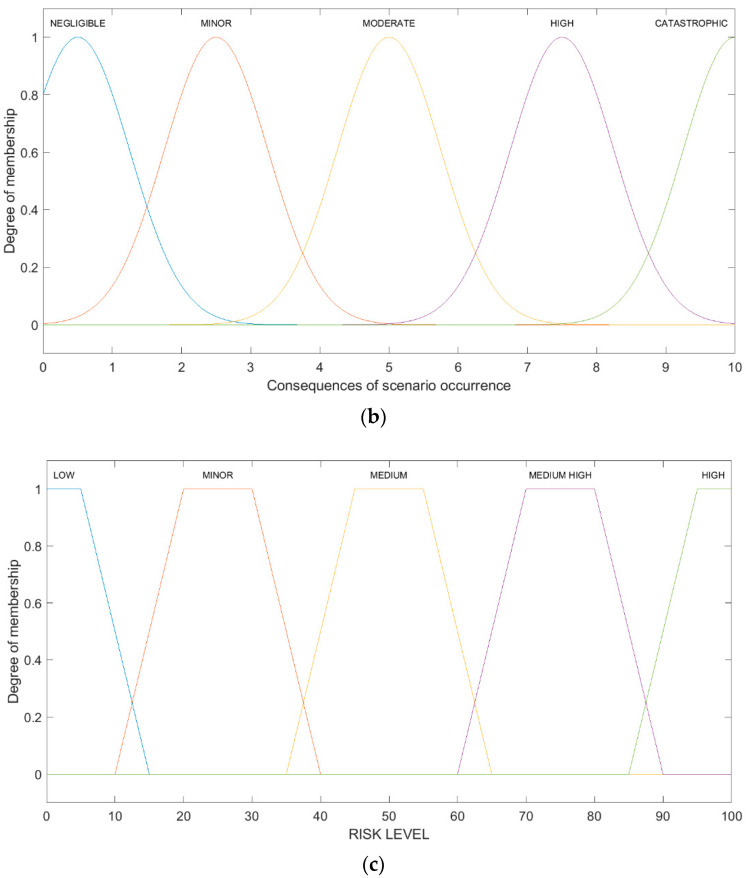
The shape of membership functions: (**a**) Likelihood of the scenario expressed by the frequency of its occurrence; (**b**) Consequences of the scenario occurrence; (**c**) Risk level.

The first step involves the fuzzification of the input parameters. Another step is to transform expert linguistic assessments into the appropriate fuzzy number. In the MAMDANI fuzzy model, the input variables are the probability and consequences of selected scenarios, and the output variable is the scenario risk level. Membership functions (Gaussian and trapezoidal) representing the linguistic scales of the input and output parameters are shown in Figure 6 (according to Table 5, Table 6 and Table 7).

The last phase of model building includes the formulation of IF-THEN rules to calculate the risk level. According to the expert knowledge in ensuring an appropriate level of safety during the inventory using drones, 25 principles have been proposed, as shown in Table 8.

**Figure 7:**
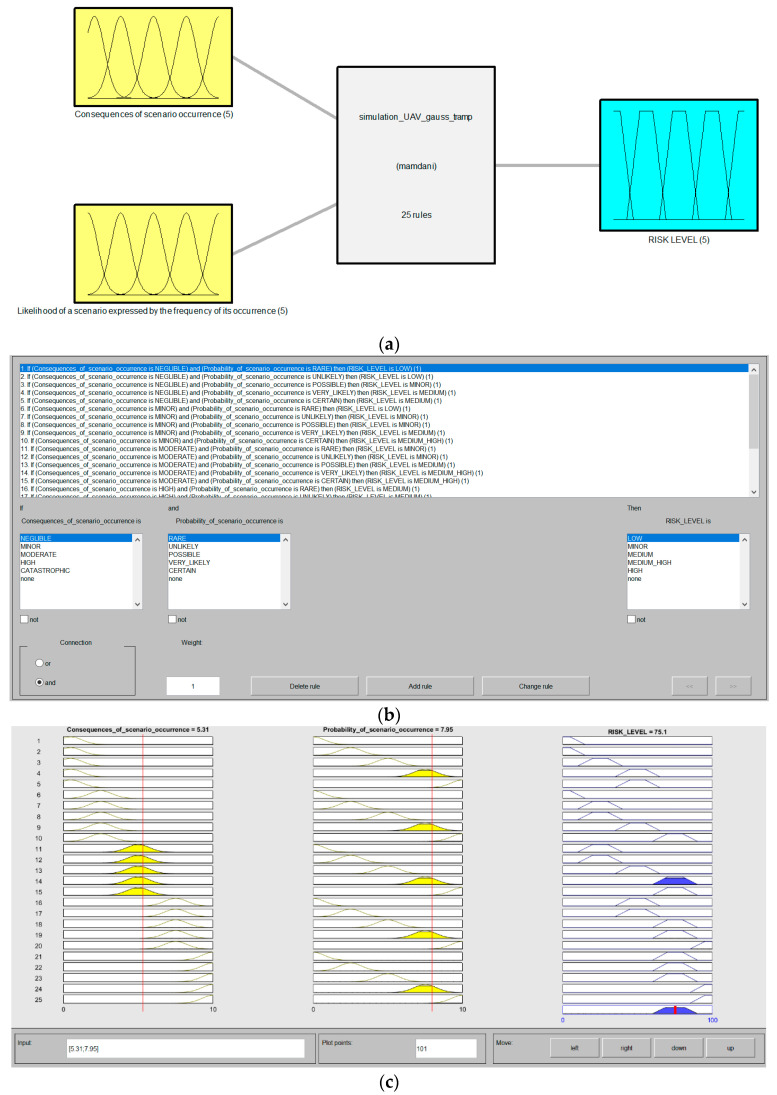
Mamdani model in MATLAB: (**a**) General view; (**b**) FIS engine with processing rules; (**c**) Rules viewer.

The fuzzy inference module (FIS), the so-called engine, analyzes all user-defined fuzzy rules, and the results of using these rules are interpreted using the MAMDANI algorithm (Figure 7). To obtain the final value of the risk level from the constructed FIS, Equation (4) is used in the process of defuzzification of the fuzzy set.

## 5. Results

The proposed methodological procedure was carried out for the scenarios and rules defined by the team of experts following the assumptions presented in Section 4. Historical data was used for the analysis, derived from measurements made during the service provision to individual clients of the enterprise. The same team of experts also evaluated the risk analysis results for the ten identified scenarios. Based on their knowledge and experience and additional information, the experts set the acceptance levels for both selected groups of scenarios: SUES and CUES. The determined level of risk acceptance allowed to indicate the appropriate attitudes of managers towards individual values of risk indicators and to define the scope of actions that should be taken concerning scenarios with unacceptable risk. All the results obtained from the conducted risk assessment procedure of the analyzed scenarios are presented in Table 9.

**Figure 8:**
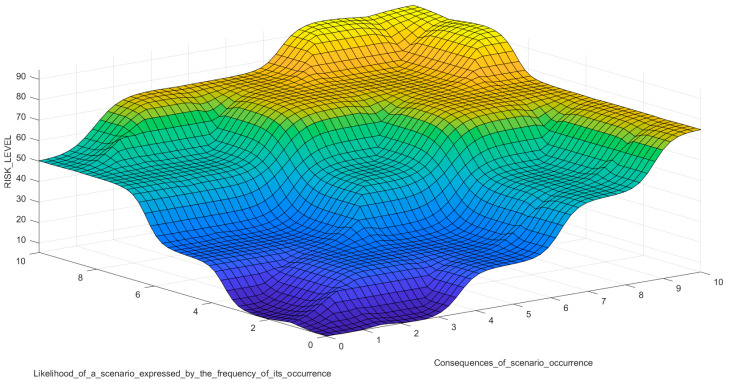
Surface risk level index for the proposed risk assessment model in MATLAB. All rules are with weight = 1.

**Figure 9:**
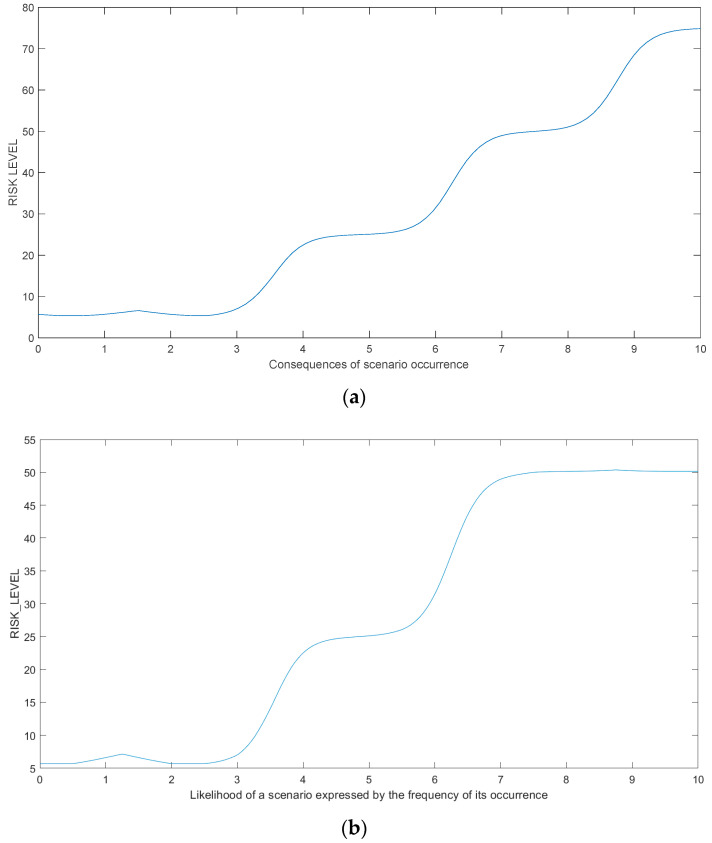
Dependencies between the input function course on the obtained risk level index: (**a**) Function of consequences of scenario occurrence; (**b**) Function of likelihood of scenario expressed by frequency of its occurrence.

To confirm the correctness of the MF input choices, apart from the risk level index surface plot (Figure 8), it also presents the dependence of individual input signals on the obtained output risk level index (Figure 9).

**Figure 10:**
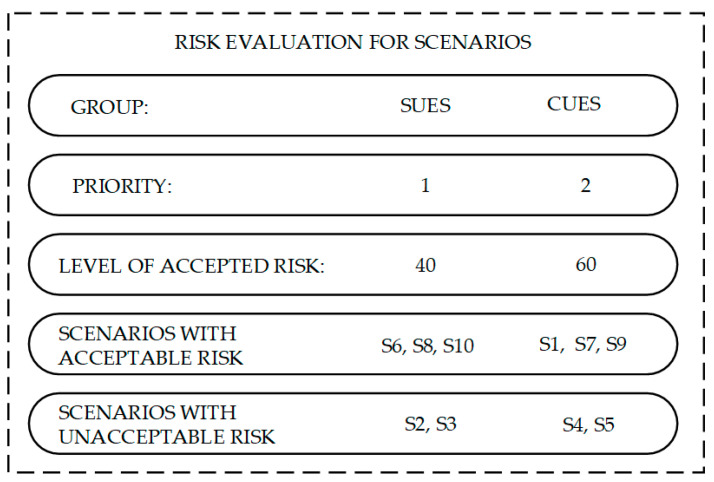
Acceptance level for the assessed scenarios in both analyzed groups.

Following the assumptions of the presented method when determining the acceptance level, the early stage of the service life cycle and the need for quick actions aimed at its improvement were taken into account. Therefore, the experts adopted a lowered acceptance threshold for the analysis to sensitize decision-makers to the risks associated with implementing a new service. In line with the proposed approach, a differentiation of the acceptance threshold was also introduced depending on the priority assigned to a given group of scenarios. Figure 10 shows the classification of scenarios, the approved point assigned to them, and the assignment of scenarios to the appropriate level. The acceptance level was determined by experts based on assessing the quality of the service provided by clients and the effectiveness of the actions taken to improve the system.

According to the results obtained, in both groups, there were two distinguished scenarios for which the risk index exceeded the acceptance threshold and three scenarios for which the risk of occurrence was considered acceptable. However, it should be noted that the risk of the S1 scenario was deemed to be acceptable because it belongs to the CUES group. In the situation of such a risk level in the SUES group, the occurrence of this scenario would be considered unacceptable.

The high priority given to the SUES group forces them to take quick and decisive actions to improve the system. The company must make immediate decisions on the direction and method of risk mitigation. In the case of both adverse events, it is necessary to modify both the system software (S2 scenario) and the mechanical structure of the device (S3 scenario). The introduced changes should therefore be aimed primarily at reducing the likelihood of this type of disturbance reoccurring.

Scenarios from the CUES group strongly depend on cooperation with the client. For this reason, preventive measures aimed at reducing the likelihood of their occurrence may not always be effective. However, they should not be abandoned for this reason. The development of procedures related to preparing the warehouse system for the service’s performance should be developed in terms of the readiness of the delivering team and the customer. However, due to the uncertainty related to the client’s proper environment preparation, it is necessary to prepare emergency scenarios that will reduce the effects of a given undesirable event. The indicated forms of risk mitigation should be undertaken concerning the S4 and S5 scenarios where the risk level exceeded the acceptable level.

In scenarios whose level of risk is acceptable, it is necessary to constantly monitor the frequency and effects of their possible occurrence. Each subsequent service performed in the client’s environment provides new data for periodically conducted risk assessment. For this reason, due to the following analytical procedures, the value of risk indicators for all scenarios may change. If the implemented improvement solutions allow reducing the level of risk for the S2, S3, S4, and S5 scenarios, in the subsequent evaluation procedure, lowering the level of acceptance should be considered to improve the service also concerning other adverse events. When lowering the level of risk acceptance, experts should, however, consider the economic rationale of the improvement decisions made.

## 6. Discussion

Experts positively verified the proposed risk assessment methodology, and its usefulness in improving the system was highly appreciated by the team providing the service to clients. A start-up was used to verify the method, which was based on the previous experiences of its co-founders when introducing a new service. This fact had a significant impact on the level of competence of the team cooperating with us in the course of the conducted research and on the openness of employees to cooperation within the framework of information exchange. Thanks to this situation, it was possible to assess the actual potential of the method and the results obtained during the evaluation procedure.

People participating in the risk assessment process emphasized the universality of the method and the uniqueness of the obtained results. They considered the key benefit of taking into account the specifics of the service provided in the first stage of introducing it to the market and the possibility of adapting the rules of the analytical procedure to the company’s requirements. A significant advantage of the proposed method is using a quantitative and qualitative approach in the analyses. In the first phase of introducing a service to the market, implementation teams often complain about insufficient historical data. This fact is given as the reason for abandoning quantitative analyses. The proposed approach partially removes this limitation. 

Despite the highly-rated usability, the company’s team members indicated that the main barrier to implementing the proposed approach in the daily assessment procedure was using a fuzzy model in MATLAB. This fact requires a person in the risk management team who has knowledge and experience handling this MATLAB module. Company team members indicated that some enterprises (especially from the small and medium-sized sector) that introduce new services to the market as a start to their business do not always have specialists who could implement the proposed approach for the service they provide. At the same time, it is worth noting that what is an advantage of the proposed method is also its limitation. Adapting the method to the specifics of the first phase of the service life cycle causes no emphasis on including data on failure, maintenance, and wear of technical equipment in the analyses. This is because new drones are used in this phase, and they are occasionally damaged. However, in the subsequent phases of their operation, the risk of their damage increases. For this reason, failure and maintenance data will play a key role in analyzing adverse events that disrupt the process of identifying goods in subsequent phases of the service life cycle. Therefore, these aspects should be the subject of detailed research in the development of the proposed method.

When assessing the methodology of the procedure, it should be noted that the proposed classification of scenarios is primary. The scenario segregation assumptions adopted in the model were primarily aimed at indicating the level of control over a given threat. According to previous Tubis research [9], identifying the level of control performed on the occurring hazards should be translated into selecting appropriate risk management methods. Two groups were introduced in the proposed methodology—their distinction results from the necessary interventions aimed at risk reduction and further service development. However, in the subsequent stages of the product life cycle, the classification of scenarios requires modification. The occurrence of disturbances as a result of an inoperative UAV system is less and less acceptable. However, additional sources of adverse events may constitute the basis for distinguishing classes of scenarios and assigning them appropriate ranks. For this reason, it is worth introducing additional methods to the cause-and-effect analysis in the first phase of risk assessment, e.g., the Ishikawa method.

Many solutions for drones in warehouse inventory use barcodes for automatic data identification [38,75]. The system assessed in this article also used this type of AID, which was necessary for the distinguished adverse event scenarios, the occurrence of which was partially related to the identification technology used. However, it should be noted that there are currently used solutions that use RFID technology in the inventory process (e.g., [33,76]). The conducted procedure for a service using this AID system would produce completely different results.

When analyzing the entire research procedure carried out, we wanted to refer to the results presented by Storey et al. [5]. In their research, this team identified front-line staff involvement as one of the main elements of market success for the new service (7th among 10 top success factors). We came to the same conclusions when implementing the proposed approach in the whole system. Members of service teams provided key information about the causes of the disturbances, and they indicated the necessary changes and improvements during the brainstorming session regarding the UAV measurement system and cooperation with the client.

## 7. Conclusions

The intangible nature of services and the simultaneous production and consumption of services impose additional requirements on the service company when introducing a new solution to the market. Unlike new product development, it is impossible to test all the conditions for its implementation in services. As a result, the success of the new service introduced to the market generates a higher level of risk than in the case of the product. As research results show, the success of the NSD is determined by various factors related to its implementation and customer service. One of such factors is the utilized potential of the first phase of its introduction to the market. This potential occurs in:

- relationships with customers and listening carefully to their comments;
- observations and collected data on the conditions and course of the service;
- implementation of risk management tools to improve the service based on experience gathered in the first stages of its implementation.

The approach to risk assessment proposed by us considers the first phase of introducing the service to the market, and the specificity of the service consisting of the use of drones in warehouse operations. Currently, we are observing the increasing popularity of the use of such systems both in traditional logistics systems and Logistics 4.0 solutions. As a result, the subject matter discussed by us can be considered current and necessary in the light of changes taking place in the business processes of enterprises. However, the universal nature of the proposed approach predisposes this methodology to be implemented in other anthropo-technical systems supporting logistic processes, e.g., using autonomous mobile robots. For this reason, further research by the authors will be focused on developing the method, taking into account the specificity of other solutions in the area of Logistics 4.0.

Thanks to the cooperation with the company, the team of researchers can observe the development of the analyzed service in subsequent phases of its life cycle. For this reason, further research is planned to analyze the changes taking place in the assessed risk in the following stages of the service life cycle. The analysis results will improve the risk assessment methodology and consider its adaptation to the cycle phase in which the service is analyzed.

## Figures and Tables

**Table 1:** Areas of application of drones in warehouses (own study based on [10]).

Warehouse Process	Application Areas
Inventory management	Inventory audit Inventory management Cycle countingItem search Buffer stock maintenance Stocktaking
Intra-logistics of items	Express delivery of tools and spare parts
Inspection and surveillance	Monitoring and inspection in dangerous areas or high altitudes Regular surveillance, for example, to prohibit theft and other unwanted behavior

**Table 2:** Examples of the risk classifications.

Authors	Internal Risk	External Risk
Christopher and Peck [44]	to the firm: process and control risks	to the network: environmental risk; to the firm but internal to the supply chain network: demand and supply risks
Wu, Blackhurst, and Chidambaram [45]	internal controllable, internal partially controllable, internal uncontrollable	external controllable, external somewhat controllable, external uncontrollable
Kumar, Tiwari, and Babiceanu [46]	demand, production and distribution, supply risks	terrorist attacks, natural disasters, exchange rate fluctuations
Olson and Wu [47]	available capacity, internal operation, information system risks	nature, political system, competitor and market risks

**Table 3:** Priorities assigned to the distinguished groups of scenarios.

Name of the Scenario Group	Group Symbol	Priority
Undesirable event scenarios for the UAV system	SUES	1
Scenarios for undesirable events resulting from the customer’s working environment	CUES	2

**Table 4:** Description of the scenarios subject to the risk assessment.

No.	Name of the Scenario	Description (Causes of Disturbances)
S1	Insufficient photo exposure	Incorrect illumination of the label when taking a photo with a camera
S2	Errors in the onboard computer software	Insufficient number of simulation tests, programming errors
S3	Vibration isolation problems (vibrations)	Incorrect vibration isolation of the mechanical structure
S4	Loss of position by the location system	Light reflections, vibrations, software errors, loss of communication between the onboard computer and the pilot, errors in the placement of aruko location markers
S5	Reflections on location markers	The technology of making location markers (aruko)
S6	Lack of communication with onboard cameras	Problems with the electrical connection/mechanical damage to the camera/improper focus in the cameras
S7	Limited bandwidth on various communication interfaces	Hardware management errors/too low (limited) hardware performance
S8	Lack of connection between onboard computer and autopilot	Electrical connection problems
S9	Lack of GCS connectivity (Ground Control Station)	Local network problems
S10	No connection to the RC (Radio controller) device	Damaged antenna, interference, broken electrical connection with the autopilot

**Table 5:** Likelihood of scenario occurrence expressed by the frequency of its occurrence.

Rating Category	Description	Fuzzy Value
CERTAIN (P5)	Expected to occur regularly under normal circumstances	(0.75, 10)
VERY LIKELY (P4)	Expected to occur at some time	(0.75, 7.5)
POSSIBLE (P3)	May occur at some time	(0.75, 5)
UNLIKELY(P2)	Not likely to occur in normal circumstances	(0.75, 2.5)
RARE (P1)	Could happen but probably never will	(0.75, 0)

**Table 6:** Consequences of scenario occurrence.

Rating Category	Description	Fuzzy Value
CATASTROPHIC (C5)	Failure to complete the mission completely, damage to the client’s property, loss of the order/client	(0.75, 10)
HIGH (C4)	Failure to complete the mission, crash or permanent damage to the drone	(0.75, 7.5)
MODERATE (C3)	Inability to start the mission, need to postpone the service to a later date	(0.75, 5)
MINOR (C2)	Disruptions causing a delay in the implementation of the service or the lack of the expected results of the mission (in terms of the quality and completeness of the measurements performed)	(0.75, 2.5)
NEGLIGIBLE (C1)	No impact or very little impact on security	(0.75, 0)

**Table 7:** Risk level.

Rating Category	Description	Fuzzy Value
HIGH (RL5)	Consequences are catastrophic or high for drone missions and the service provided, which occurs certainly or almost certainly in the future	(85, 95, 100, 100)
MEDIUM HIGH (RL4)	A certain (almost certain) event with minor consequences or an unlikely event with critical consequences	(60, 70, 80, 90)
MEDIUM (RL3)	An almost certain event with minor consequences or an unlikely event with significant consequences Limiting the possibility of a successful drone mission	(35, 45, 55, 65)
MINOR (RL2)	The occurrence of an event is possible—minor/no significant consequences without much impact on the mission’s success	(10,20, 30, 40)
LOW (RL1)	It is almost impossible for an event to occur. The consequences are negligible—no impact on the accomplishment of the mission	(0, 0, 5, 15)

**Table 8:** Risk decision matrix.

	C1	C2	C3	C4	C5
**P1**	LOW	LOW	MINOR	MEDIUM	MEDIUM
**P2**	LOW	MINOR	MINOR	MEDIUM	MEDIUM HIGH
**P3**	MINOR	MINOR	MEDIUM	MEDIUM HIGH	MEDIUM HIGH
**P4**	MEDIUM	MEDIUM	MEDIUM HIGH	MEDIUM HIGH	HIGH
**P5**	MEDIUM HIGH	MEDIUM HIGH	MEDIUM HIGH	HIGH	HIGH

**Table 9:** Results of empirical experiments and risk score for all considered scenarios. All rules are with weight = 1.

Scenario	Occurrence Probability Based on Historical Research Results during Services Provided to Clients (%)	P (Fuzzy)	P (Crisp)	C (Fuzzy)	C (Crisp)	Risk Score
**S1**	18.54	P4	7.72	2.59	C2	50.3
**S2**	16.10	P4	7.77	5.89	C3	75.1
**S3**	15.12	P4	7.97	7.22	C4	75.3
**S4**	14.15	P3	4.82	7.56	C4	74.5
**S5**	10.24	P3	5.31	7.95	C4	75.1
**S6**	8.78	P3	4.99	2.33	C2	25.2
**S7**	5.85	P2	2.87	2.54	C2	25.1
**S8**	4.88	P2	2.33	5.11	C3	25.3
**S9**	4.39	P2	2.56	5.54	C3	26.6
**S10**	1.95	P2	2.56	5.33	C3	25.9

## Data Availability

The data presented in this study are available on request from the corresponding author. The data are not publicly available due to the fact that the data are the property of the authors.
